# Identification and Expression Analysis of Candidate Genes Associated with Defense Responses to *Phytophthora capsici* in Pepper Line “PI 201234”

**DOI:** 10.3390/ijms160511417

**Published:** 2015-05-18

**Authors:** Pingyong Wang, Xiaodan Liu, Jinju Guo, Chen Liu, Nan Fu, Huolin Shen

**Affiliations:** Beijing Key Laboratory of Growth and Developmental Regulation for Protected Vegetable Crops, China Agricultural University, Beijing 100193, China; E-Mails: wpy0320fn@cau.edu.cn (P.W.); lxd123@cau.edu.cn (X.L.); gjj1987cool@cau.edu.cn (J.G.); liuchen@cau.edu.cn (C.L.); funan@cau.edu.cn (N.F.)

**Keywords:** RNA-seq, *Capsicum annuum*, *Phytophthora capsici*, defense response, gene expression

## Abstract

*Phytophthora capsici* (Leonian), classified as an oomycete, seriously threatens the production of pepper (*Capsicum annuum*). Current understanding of the defense responses in pepper to *P. capsici* is limited. In this study, RNA-sequencing analysis was utilized to identify differentially expressed genes in the resistant line “PI 201234”, with 1220 differentially expressed genes detected. Of those genes, 480 were up-regulated and 740 were down-regulated, with 211 candidate genes found to be involved in defense responses based on the gene annotations. Furthermore, the expression patterns of 12 candidate genes were further validated via quantitative real-time PCR (qPCR). These genes were found to be significantly up-regulated at different time points post-inoculation (6 hpi, 24 hpi, and 5 dpi) in the resistant line “PI 201234” and susceptible line “Qiemen”. Seven genes were found to be involved in cell wall modification, phytoalexin biosynthesis, symptom development, and phytohormone signaling pathways, thus possibly playing important roles in combating exogenous pathogens. The genes identified herein will provide a basis for further gene cloning and functional verification studies and will aid in an understanding of the regulatory mechanism of pepper resistance to *P. capsici.*

## 1. Introduction

*Phytophthora capsici* (Leonian), classified as an oomycete, is a soil born pathogen that can infect pepper foliage, fruits, stem, and roots causing a significant reduction in pepper yields and quality. Root rot is one of the most serious disease symptoms, which can result in plant wilting and death when plants are infected with *P. capsici* [1]. The spread of *P. capsici* is accelerated by high temperatures and humidity and is managed through cultural practices, fungicide applications and the use of resistant cultivars [2]. However, these methods are only partly effective, with metalaxyl insensitivity reported in both *P. capsici* laboratory and field experiments [3,4]. This makes the utilization of genetically resistant cultivars a promising and environmentally friendly control method. Thus, the selection of cultivars with higher resistance levels has become of major interest for plant breeders [5]. USDA “PI 201234” from Central America, “Criollode Morelos 334” (“CM 334”) from Mexico and “Perennial” from India are highly resistant to *P. capsici* [6]. Using these resources, breeders have cultivated several commercial cultivars, but none of them possess broad resistance to this pathogen.

Reports examining the inheritance of *P. capsici* resistance in peppers are variable. The reported genetic models of PI 201234 included one dominant gene [7,8], and a single dominant gene with modifiers [9]. In “CM 334”, studies have reported two recessive genes, two dominant genes, three genes and an additive gene, or polygenes with additive or epistatic effects [1,10,11,12,13]. A polygenic system is also seen in the “Perennial” line, with additive and epistatic effects noted. These reports all indicate that the regulatory mechanisms of pepper *P. capsici* resistance are complex.

Based on these genetic models, different quantitative trait loci (QTL) have been reported, with the most consistent results indicating that the QTLs on chromosome P5 have major roles in pepper *P. capsici* resistance [6,13,14,15,16,17,18,19,20,21]. Recently, *CaDMR1*, a candidate gene that encodes a homoserine kinase, was reported to be highly associated with the major QTL *Pc5.1* [22]. Additionally, a single nucleotide polymorphism marker (SNP) on chromosome 5, Phyto5NBS1, was reported to be highly associated with resistant/susceptible traits against low virulence *P. capsici* strains [23].

In recent years, RNA-seq technology has been widely used to provide precise gene expression measurements by mapping short reads to a reference genome [24,25,26,27,28]. In this study, Illumina paired-end sequencing technology was used to analyze root transcriptome changes in the highly resistant “PI 201234” line following *P. capsici* inoculation. The sequencing results and the expression patterns of some differentially expressed genes (DEGs) were further validated using qPCR. Overall, these findings will aid in understanding pepper defense mechanisms against *P. capsici*.

## 2. Results

### 2.1. Sequencing Output and Mapping Reads to the Genome

**Figure 1:**
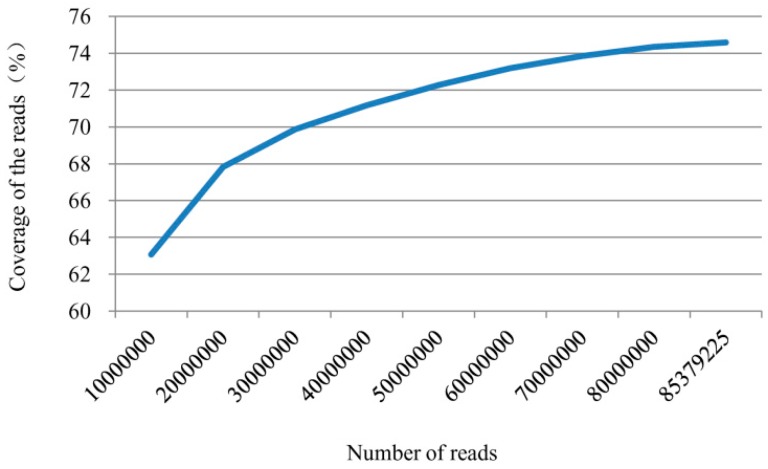
Coverage of all high quality reads from two libraries to *Zunla-1* coding DNA sequences.

To identify genes involved in the pepper defense responses to *P. capsici*, library A (inoculated with zoospore suspension) and library CK (control) were constructed. Each library generated 2 × 100 bp reads from either end of a DNA fragment via Illumina paired-end sequencing. In the present study, a total of 79,250,598 and 75,339,602 raw reads (100 bp in length) were obtained from libraries A and CK. After a strict quality assessment and data cleaning, 76,015,888 (95.92%) and 72,130,988 (95.74%) high quality reads were obtained. The retained high quality reads were then mapped to the pepper genome (*Zunla-1*) using TopHat, with 71,904,454 (94.60%) reads in library A and 67,803,241 (94.0%) reads in library CK successfully mapped to the *Zunla-1* pepper genome. A total of 85,379,225 high quality reads from the two libraries could cover 74.59% of the DNA coding sequences (Table 1 and Figure 1).

**Table 1:** Summary of sequencing and assembly results. Library A: root sample from pathogen-inoculated plants; Library CK: root sample from water-inoculated plants.

Class	Library A			Library CK		
	Number	Total Length (bp)	Percentage (%)	Number	Total Length (bp)	Percentage (%)
Raw reads	79,250,598	7,925,059,800		75,339,602	7,533,960,200	
Clean reads	76,015,888	7,152,478,994	95.92	72,130,988	6,782,519,810	95.74
Mapping to genome	71,904,454		94.60	67,803,241		94.00
Total mapping position	86,610,410			86,962,176		

**Figure 2:**
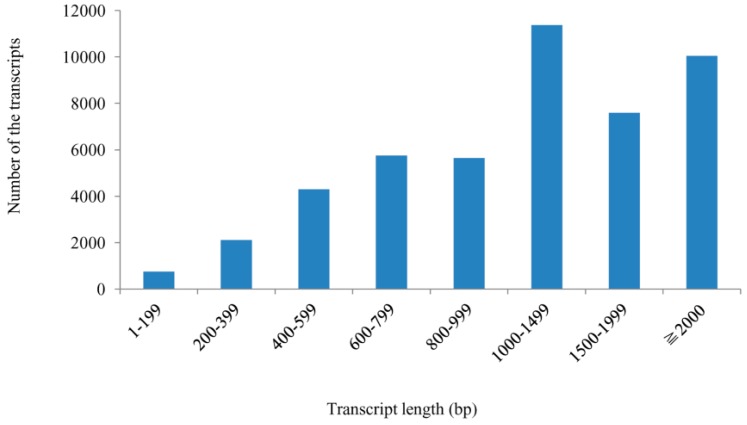
Length distribution of all assembled transcripts within the two libraries.

All high quality reads were assembled using the Cufflinks software, with 47,575 non-redundant transcripts obtained with an average length of 1437.22 bp and N50 (the median transcript length) of 1789 bp. Of those transcripts, 84.9% were more than 600 bp, 61% were more than 1000 bp, and 21.1% were more than 2000 bp (Figure 2).

All of the 47,575 transcripts could be anchored to 30,106 gene loci within the reference genome, with 29,262 (64%) being known loci and 10,844 (36%) being novel. Among these genes, 118 were expressed only in library A and 102 only in library CK, while 29,870 genes were expressed in both libraries (Table 2).

**Table 2:** Gene expression statistics for the two libraries.

Class	Number	Percentage (%)
Total genes	30,106	100
Expressed genes	30,090	99.95
Expressed in library A	29,988	99.66
Expressed in library CK	29,972	99.61
Expressed both	29,870	99.27
Expressed only in library A	118	0.39
Expressed only in library CK	102	0.34

### 2.2. Kyoto Encyclopedia of Genes and Genomes (KEGG) Functional Classifications

**Figure 3:**
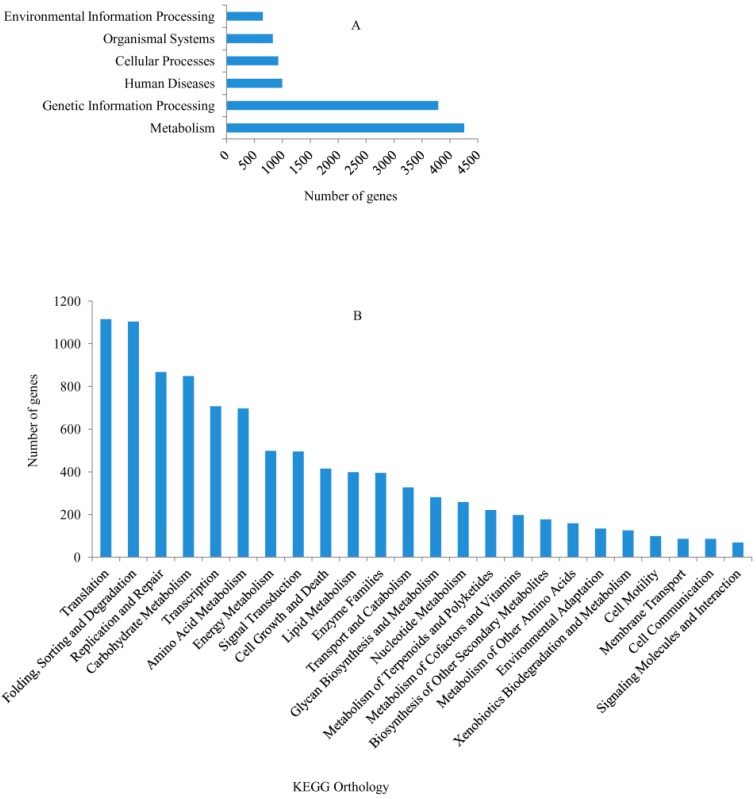
Kyoto Encyclopedia of Genes and Genomes (KEGG) pathway assignments. (**A**) Classifications based on metabolic categories; (**B**) KEGG subcategory classifications.

Out the 30,106 genes, 21,976 (73.0%) had significant matches and 5168 (23.52%) were annotated via the Kyoto Encyclopedia of Genes and Genomes (KEGG) database. Among the 5168 annotated genes, 4255 were anchored to metabolic pathways, making it the largest KEGG classification group (Figure 3A). Moreover, with the exception of human disease pathway, other pathways could also be classified into 24 subcategories, with the largest classifications being translation (1115 genes), folding, sorting and degradation (1104 genes), replication and repair (867 genes) and carbohydrate metabolism (848 genes) (Figure 3B). Overall, these genes involved in signal transduction, lipid metabolism, glycan biosynthesis and metabolism, metabolism of terpenoids and polyketides, biosynthesis of other secondary metabolites, environmental adaptation and membrane transport may be related to plant-pathogen interactions.

### 2.3. Functional Classifications via Interpro and Gene Ontology (GO)

**Figure 4:**
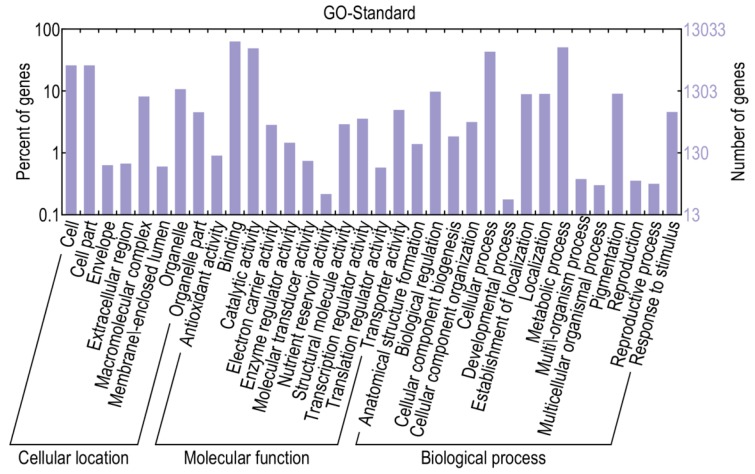
Gene ontology classifications of assembled transcripts.

Based on sequence homology, 13,040 genes could be assigned to at least one of the three gene ontologies (cellular location, molecular function, and biological process) at *E*-value ≤ 1 × 10^−10^. Genes from the two libraries were further classified into 34 functional subcategories, with cell (3363 genes), cell part (3363 genes), binding (8172 genes), catalytic activity (6331 genes), metabolic process (6573 genes), and cellular process (5590 genes) being dominant among the subcategories (Figure 4). Based on the annotations, 593 genes were classified into the category “response to stimulus”, 462 genes into “transcription regulator activity” and 117 genes into “antioxidant activity”, with these genes more likely to be involved in defense responses.

### 2.4. Identification and Annotation of Potential Differentially Expressed Genes

**Figure 5:**
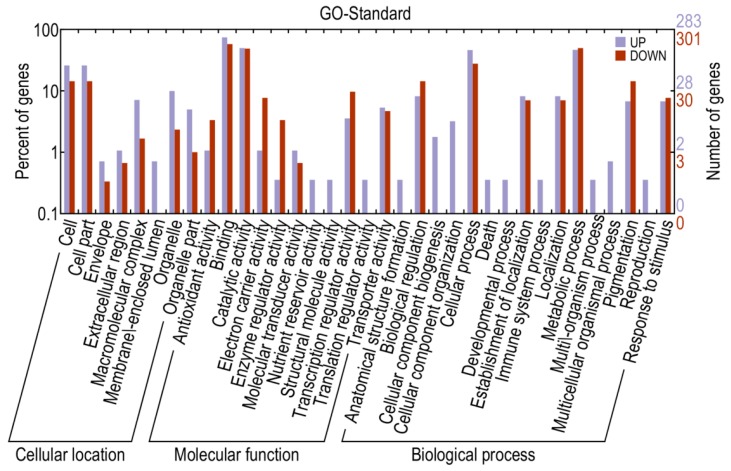
Gene ontology classifications for differentially expressed genes.

The results indicated that 14,216 genes were up-regulated, while 15,874 were down-regulated, in library A relative to library CK. Using a corrected *p*-value ≤0.05 and log_2_ (fold change) ≥1 or ≤−1 as the threshold, 1220 DEGs were identified, including 480 up- and 740 down-regulated genes which may be associated with pepper defense responses against *P. capsici* (Table S1). When compared with KEGG, 956 DEGs were annotated and 168 of them were assigned to KEGG pathways (Table S2). When compared to the InterPro database, 699 DEGs were annotated, while 636 DEGs were annotated via the gene ontology (GO) database. More specifically, 522 DEGs were mapped to the molecular function category, 119 to the cellular component category and 369 to the biological process category (Figure 5 and Table S3). Most of the genes categorized under molecular function were involved in binding and catalytic activity, while a large numbers of genes categorized under cellular component were classified to “cell” and “cell part”. As for DEGs categorized under biological process, genes related to metabolic processes, cellular process, biological regulation, pigmentation, localization, establishment of localization and response to stimulus were significantly triggered. Furthermore, many genes mapped to “antioxidant activity”, “transcription regulator activity” and “response to stimulus” were highly differentially expressed between resistant and susceptible lines, thus possibly associated with pathogen defense.

Of the detected genes, 211 potential defense response genes were identified, to include genes involved in pathogen-associated molecular patterns, effector-triggered immunity, ion fluxes, plant hormones biosynthesis, and signaling, transcription factors, oxidative burst responses, pathogenesis-related proteins, phytoalexins biosynthesis, programmed cell death, cell wall modification, and the ubiquitin system. Additionally, some of the annotated DEGs included UDP-glucosyltransferase, cytochrome P450 and lectin (Table S4).

### 2.5. RNA-Seq Validation and Selection of Potential Defense-Related Genes

**Figure 6:**
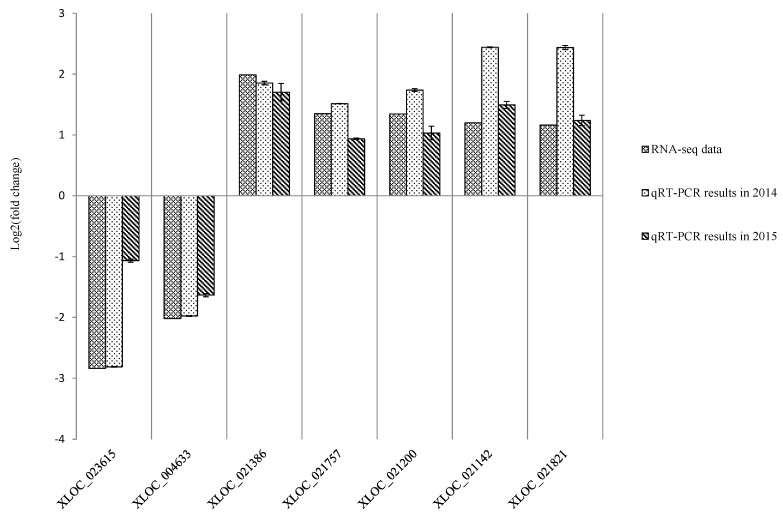
Expression pattern validation of selected genes via qPCR. The expression profiles for seven selected genes were examined via qPCR. The *Y*-axis displays transcript fold changes (log_2_) and the bars of RNA-seq data indicate transcript abundance changes calculated by the FPKM method (Fragments Per Kilobase of exon per Million mapped reads). The bars with associated standard error bars represent relative expression levels as determined by qPCR using the 2^−ΔΔ*C*t^ method. Results represent a mean ± SD of three biological replicates.

To evaluate the validity of the Illumina analysis, seven genes were selected and examined in both the A and CK libraries via qPCR (Table 3). The experiment was performed in 2014 and repeated in 2015. The qPCR expression profiles were in general consistent with the RNA-seq findings, suggesting the RNA-seq data was reliable (Figure 6).

**Figure 7:**
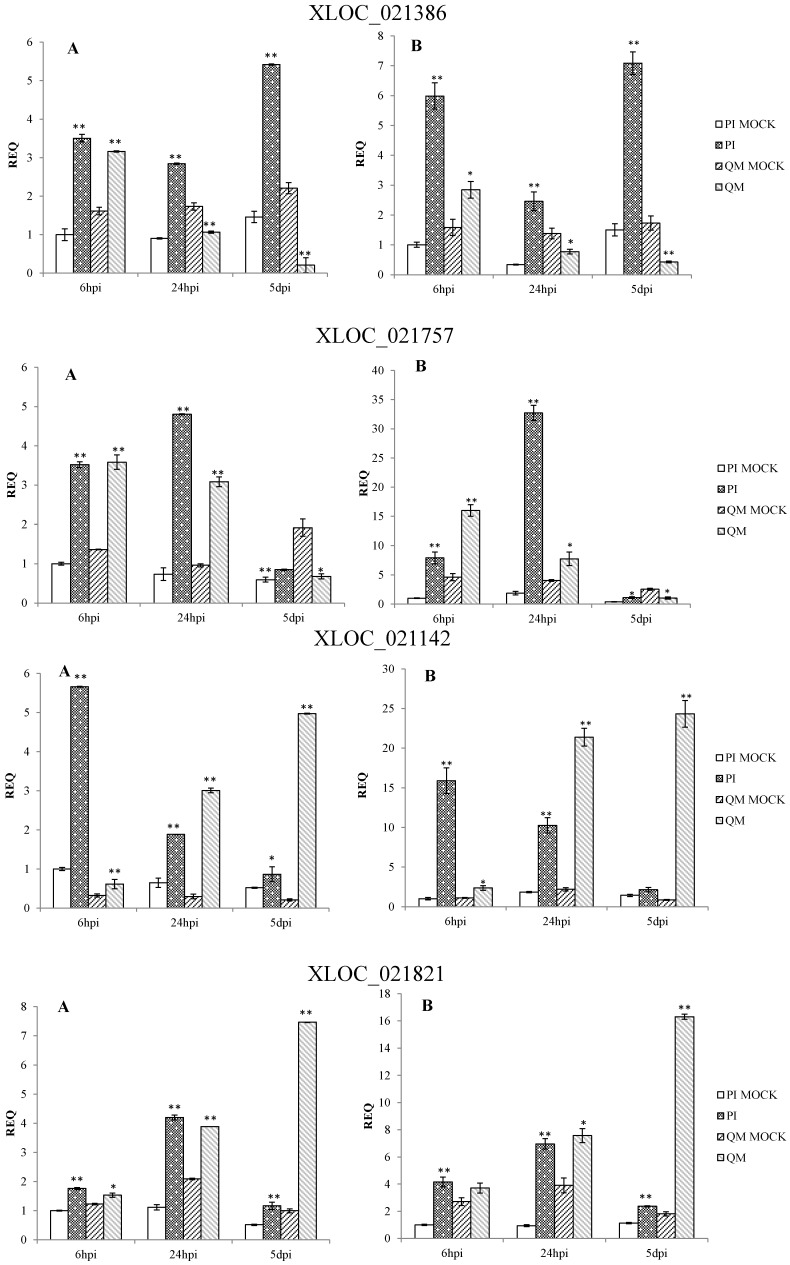

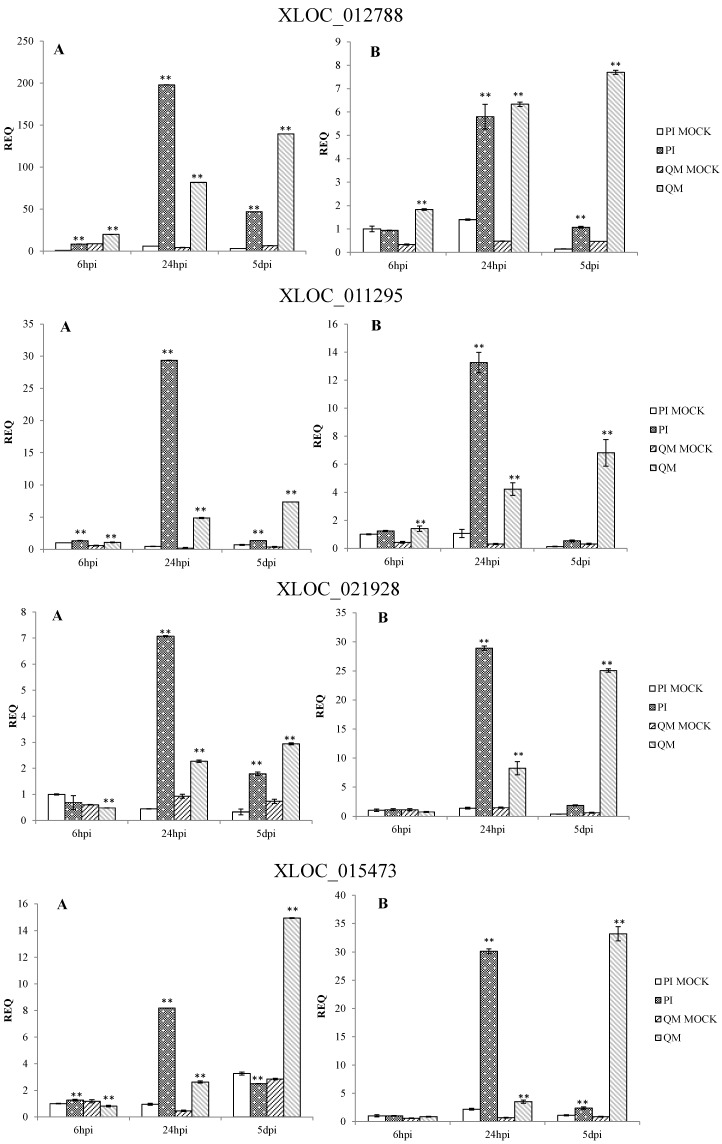

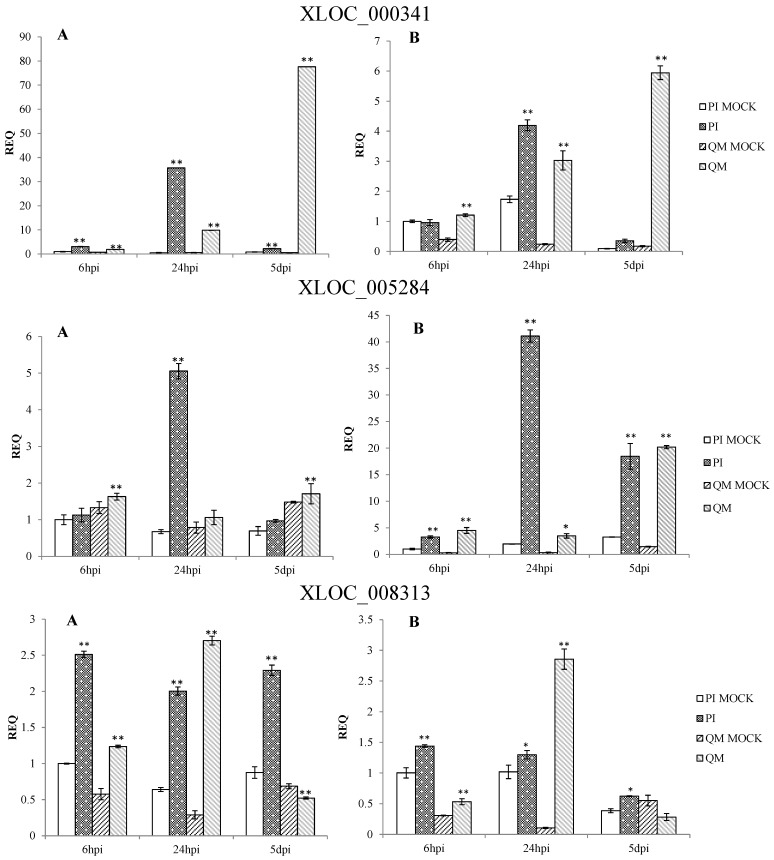

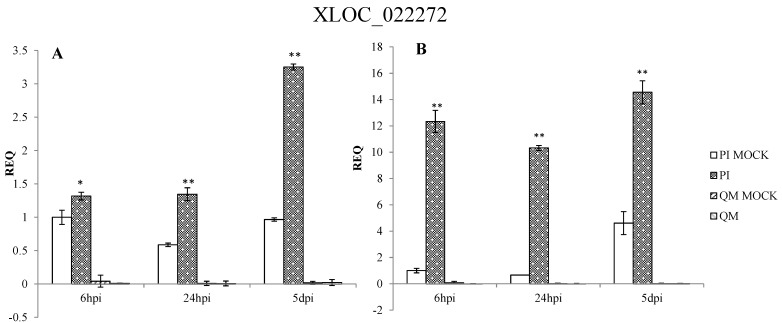
qPCR analysis of 12 differentially expressed genes in pepper. Two independent experiments were performed in the year 2014 (**A**) and 2015 (**B**), respectively. PI: “PI 201234” inoculated with *P. capsici* zoospore suspension; PI MOCK: “PI 201234” inoculated with sterile-distilled water; QM: “Qiemen” inoculated with *P. capsici* zoospore suspension; QM MOCK: “Qiemen” inoculated with sterile-distilled water. Gene expression was normalized to *actin* with the data displayed as a mean ± SD of three biological replicates and an asterisk indicating statistical significance between infected and corresponding mock treatments. *T*-test was performed at different levels of significance. One asterisk represents that the differences reach 0.05 level of significance and two asterisks represent the differences reach 0.01 level of significance.

qPCR results confirmed that the expression patterns of 12 DEGs in the two independent experiments were consistent. These DEGs were significantly up-regulated at different time points post-inoculation in the resistant line “PI 201234” and susceptible line “Qiemen”, but in differing intensities. (Table S5 and Figure 7). Based on the functional annotations, these genes are related to defense response processes such as ethylene signaling (*XLOC_005284*, *XLOC_021821*, *XLOC_021142*), jasmonic acid (JA) signaling (*XLOC_015473*), peroxidase (*XLOC_012788*, *XLOC_021757*), phytoalexins synthesis (*XLOC_011295*, *XLOC_000341*), bulb-type lectin (*XLOC_022272*), cell detoxification (*XLOC_021928*, *XLOC_021386*) and WRKY transcription factor (*XLOC_008313*).

**Table 3:** qPCR primers used to validate RNA-seq data.

Gene Name	Reference Gene	Annotation	Primer (5'–3')
*XLOC_023615*	*Capana06g000792*	SWEET sugar transporter	F: ATTGCTCCAAAGCCACCACC
			R: TGGCAGCATCGTCTCGTTCA
*XLOC_004633*	*Capana01g001100*	Major intrinsic protein, conserved site	F: TTGTGGCTGTTTCAGTGTCA
			R: GGTAGCAATCTTGAGGAGGA
*XLOC_021386*	*Capana05g000172*	Proteinase inhibitor I25, cystatin, conserved region	F: AGGCGAAGACAAATCTGGAAT
			R: TGCTAAATAGTTATGTGGCGAGTC
*XLOC_021757*	*Capana05g001613*	Plant peroxidase	F: GTATTACTCGGCAGAAGGGACTC
			R: GTGGTTGGGCTTGTGGTGT
*XLOC_021200*	*Capana05g002178*	Plant peroxidase	F: CTTTTCCACGATTGTTTTGTTAGG
			R: CGACCTGCTGGCACTGAAT
*XLOC_021142*	*Capana05g001951*	AP2/ERF domain	F: TCCTCATACCTAAACGAACCCA
			R: AGTTGTTGTCGTGTGTTGGATTG
*XLOC_021821*	*Capana05g001948*	AP2/ERF domain	F: TTGAAAGAATCTCGGACACCC
			R: GAAATTGAACGGCGACCAG

Following pathogen inoculation, seven DEGs (*XLOC_021757*, *XLOC_021821*, *XLOC_012788*, *XLOC_011295*, *XLOC_021928*, *XLOC_015473* and *XLOC_000341*) were up-regulated in “PI 201234”, with the highest expression levels reached at 24 hpi. However, in “Qiemen”, six of the DEGs were significantly up-regulated, with the highest level reached at 5 dpi, while *XLOC_021757* expression peaked at 6 hpi. At 24 hpi, *XLOC_005284* expression peaked in “PI 201234” but at 6 hpi and 5 dpi it showed a substantially lower fold change. In “PI 201234”, *XLOC_021142* showed a gradually decreased expression level with time, while the opposite was seen in “Qiemen”. Also following pathogen infection, *XLOC_008313* expression in “PI 201234” was up-regulated at three time points; while in “Qiemen”, its expression peaked at 24 hpi but was quite low during the other two time points. In contrast to the other DEGs, *XLOC_022272* was up-regulated in “PI 201234” post-inoculation, but was expressed at a much lower level in “Qiemen” throughout.

## 3. Discussion

### 3.1. RNA-Seq Dataset Analysis

About 90% of the raw sequencing reads and all of the assembled transcripts were mapped to the pepper genome. Moreover, the sequencing results were corroborated via qPCR to show that the RNA-seq data was accurate and reliable. In this study, all of the transcripts were anchored to 30,106 pepper gene loci, with 10,844 (36%) loci being novel. The transcripts anchored to the novel loci could greatly improve annotations for the existing genome and reveal more information about transcriptional and post-transcriptional regulation [29].

### 3.2. Potential Defense Related Genes against P. capsici

When infected by a pathogen, plants will perform a series of timely and effective defense responses to include cell wall modification, phytoalexin and antibiotic protein biosynthesis and programmed cell death to effectively inhibit pathogen expansion [30]. In the present study, 1220 differentially expressed genes were explored using RNA-seq, with many of these DEGs involved in defense responses and thus providing a basis for further study.

#### 3.2.1. Cell Wall Modification

In plants, the cell wall serves as a protective barrier against pathogen penetration. In this study, expression levels of plant lipid transfer protein [31,32], glutathione *S*-transferase [33,34], callose synthase [35,36,37,38,39] and lignin-forming anionic peroxidase [40,41,42] were significantly affected during plant-pathogen interactions post-infection. These proteins acted at different steps of cutin, callose and lignin biosynthesis. For example, *XLOC_012788*, a gene encoding a type of lignin-forming anionic peroxidase, displayed a much higher fold-increase at 24 hpi in PI 2012334 than in “Qiemen”, with levels not peaking in “Qiemen” until 5 dpi. These findings suggest that this gene exhibits a timely response during a pathogenic infection in “PI 201234” and therefore may contribute to the defense response.

#### 3.2.2. Phytoalexins

Phytoalexins are secondary metabolites that are induced by stress and possess antimicrobial activity against a great variety of pathogens [43]. Different kinds of phytoalexins have been reported in peanut [44], soybean [45], sorghum [46], grapevine [47], rice [48], maize [49], and pepper [50]. Furthermore, terpenoid phytoalexins, such as kauralexins, zealexins, and capsidiol [43], can prevent the germination and growth of several fungal species. In this study, both *XLOC_011295* and *XLOC_000341* had similar expression patterns, were annotated as terpene synthases and were highly expressed in “PI 201234” at 24 hpi. These findings suggest that at 24 hpi, “PI 201234” was putting up a fierce pathogenic defense.

#### 3.2.3. Phytohormones

As a kind of signaling molecule, phytohormones play an important role in biotic stress responses. Ethylene is an important signaling molecule involved in regulating defense responses in plants [51,52]. As previously reported, the application of exogenous ethylene can induce defense responses to *P. capsici* in susceptible habanero peppers, which is in agreement with other reports regarding the positive effect of ethylene on the resistance of *Solanumly copersicum* to *P. capsici* [53,54]. Furthermore, AP2-like ethylene-responsive transcript factor (AP2/ERF) is important in response to tissue damage and the development of pathogenic symptoms [55]. In *Arabidopsis*, over-expression of the ERF genes *Pti4* and *Pti5* from tomato conferred resistance to *Pseudomonas syringae* and *Erysiphe orontii* [56,57]. In the present study, three genes (*XLOC_005284*, *XLOC_021821* and *XLOC_021142*) with homology to AP2/ERF were identified and presented different levels of up-regulation in both the resistant line “PI 201234” and susceptible line “Qiemen”. Following pathogen inoculation, “PI 201234” showed no disease symptoms during the time course while “Qiemen” displayed necrotic root regions at three dpi, possibly due to the differential up-regulation of these genes in the two pepper lines.

Previous studies have indicated that JA acts as a key signal molecule during the activation of plant immune responses to necrotrophic microorganisms [58]. JA signal transduction is mediated by JA biosynthesis, jasmonate-zim domain (JAZ) ubiquitination and MYC2 transcription factor activation [59]. JAZs act as the link of the up- and down-stream of JA signaling transduction pathway. In this study, a JAZ-like gene (*XLOC_015473*) was significantly up-regulated at 24 hpi in both lines. And at 5 dpi, this gene appeared to be rapidly up-regulated in “Qiemen”. These findings suggest that the JAZ-like gene may play important roles in altering JA signaling and disease symptom development as reported in *Arabidopsis thaliana* [60].

#### 3.2.4. Analysis of a Novel Gene on Chromosome 5 in the *Zunla-1* Genome

Recent reports have suggested that the major resistance QTL is located on chromosome 5 [22,23] and that *CaDMR1* is highly associated with this QTL, but functional validation has not been reported. Among the 12 examined DEGs (Figure 7), six DEGs (*XLOC_021142*, *XLOC_021386*, *XLOC_021757*, *XLOC_021821*, *XLOC_021928* and *XLOC_022272*) were on chromosome 5. *XLOC_022272*, which maps between the 170677412 and 170678655 genomic positions of chromosome 5 (P5), encodes a bulb-type lectin and matches none of the reference genes. It was up-regulated post *P. capsici* infection in “PI 201234”, while its expression levels were much lower in “Qiemen” throughout. Previous studies have suggested that plant lectins, a group of carbohydrate-binding proteins, could act as physical barriers against pathogenic invasion by binding glycoconjugates present on the surfaces of microorganism [61]. Furthermore, interactions between endogenous proteins and exogenous carbohydrates could be an important defense signal and regulate a series of defense responses during a pathogenic infection [62]. This gene will require further examination via gene cloning, functional assays and silencing experiments to provide more insight into its role in defense.

## 4. Experimental Section

### 4.1. Biological Material for RNA-Seq

“PI 201234”, a *C. annuum* line that is resistant to *P. capsici*, was selected for sequencing. Seeds were sown in plastic cells measuring 9 × 7 × 8 cm with one seed per cell and grown in a growth chamber in the Shangzhuang experimental station at the China Agricultural University. The temperature was maintained at 28/16 °C (day/night) and the photoperiod was 16 h light/8 h dark. The tested plants were watered every other day.

### 4.2. Biological Material for Selection of Candidate Genes

The resistant line, “PI 201234”, and susceptible line, “Qiemen”, were used to further analyze the RNA-seq gene expression findings. The materials were grown under the same environmental conditions stated above.

### 4.3. Inoculation Procedure and Sample Collection

*P. capsici* isolate collected from China was provided by Xili Liu (China Agricultural University) and was identified as physiological race 2. The culture of *P. capsici* hyphae and the obtainment of zoospore suspension were according to the method described by Pang *et al.* [63]. The zoospore suspension was filtered with two layers of gauze and the number of zoospores was counted with a hemocytometer and diluted to 1 × 10^5^ zoospores per mL [15].

Before inoculation, test plants were flooded with water. Inoculation was carried out on seedlings with six leaves by releasing 1 mL zoospore suspension into the soil of each plant. Inoculated plants were kept at 80% ± 10% relative humidity and 28 ± 2 °C under 10,000 lx illumination for 16 h/day. Controls were mock inoculated with sterile-distilled water.

Root samples for sequencing were collected at 6 h, 24 h, and 5 day post-inoculation in triplicate for each time point, with control samples collected at the same time. All samples were washed clean with distilled water, wrapped in tinfoil and immediately snap-frozen in liquid nitrogen and stored at −80 °C until further use.

### 4.4. RNA Extraction and Library Preparation for Transcriptome Sequencing

“PI 201234” triplicate root samples were pooled and total RNA was extracted at 6 hpi, 24 hpi, and 5 dpi from the pathogen-inoculated and control plants using TRIzol reagent according to the manufacturer’s instructions (Invitrogen, Carlsbad, CA, USA). Residual DNA was removed using RNase-free DNaseI (Fermentas, Waltham, MA, USA) and the RNA was quantified using NanoDrop (Thermo Scientific, Waltham, MA, USA) and qualified using the RNA 6000 Pico LabChip on an Agilent 2100 Bioanalyzer (Agilent, Palo Alto, CA, USA). The pooled RNA from the pathogen-inoculated and control plants were used to construct sequencing libraries A and CK respectively.

mRNA was enriched using a mRNA purification kit (Promega, Fitchburg, WI, USA) according to the manufacturer and reverse-transcribed using Powerscript™ II (Takara, Katsushika, Tokyo, Japan) with specific primers. Next, double strand cDNA was amplified using the SMART™ cDNA Library Construction Kit and purified with a DNA purification kit (Qiagen, Hilden, Germany) to generate two high quality cDNA libraries. Approximately 10 μg of sheared cDNA from the two libraries were prepared for sequencing. After adapter ligation and agarose gel separation, the libraries were constructed using 300–500 bp fragments and were paired-end sequenced on the Illumina Genome Analyzer (Illumina Inc., Santiago, CA, USA). The sequencing raw reads have been deposited into the NCBI SRA database (http://www.ncbi.nlm.nih.gov/sra/), with accession numbers being SRR1930139 for library A and SRR1930150 for library CK.

### 4.5. Transcriptome Data Processing and Assembly

After removing low quality reads, the retained high quality reads were mapped to the pepper genome (*Capsicum.annuum*.L_*Zunla-1*, http://peppersequence.genomics.cn) using TopHat with default parameters and assembled using Cufflinks [64,65] to construct unique transcripts.

### 4.6. Functional Annotation and Classification

Functional annotations were performed by comparing sequences with public databases. All genes were compared with the Kyoto Encyclopedia of Genes and Genomes database (KEGG, release 58) [66] through BLASTX with an E-value ≤1 × 10^−10^. Pathways were established based on KO (KEGG Orthology) information retrieved by a Perl program. Gene domains [67] were predicted by InterProScan (release 4.8) and functional assignments were mapped to Gene Ontology (GO) (http://www.geneontology.org/) [68]. GO classifications and a GO phylogenetic tree were obtained using WEGO (http://wego.genomics.org.cn/cgi-bin/wego/index.pl) [69].

### 4.7. Detection of Differentially Expressed Genes

Cuffcompare was used to compare the assembled transfrags for each library to the reference annotation to build non-redundant transcript data sets. Then Cuffdiff was used to find significant changes in gene expression levels [70]. Finally, the genes were defined as differentially expressed genes only when the threshold values were log_2_ (fold change) ≥1 or ≤−1 and *p*-value ≤ 0.05.

### 4.8. RNA-Seq Validation via qPCR

Seven DEGs were randomly selected from the RNA-seq data for validation via qPCR. Appropriate primers were designed and cDNAs were reverse transcribed from 2 µg of total RNA using a cDNA synthesis kit (6210A, Takara, Katsushika, Tokyo, Japan). qPCR was performed on an ABI PRISM 7500 real-time PCR System (Applied Biosystems, Waltham, MA, USA) according to the instructions of the SYBR Premix Ex Taq™ Kit (RR420A, Takara). The pepper *actin* gene (GenBank: GQ339766.1) was used as an internal control gene and expression levels were analyzed using the 2^−ΔΔ*C*t^ method. The experiment was performed in 2014 and repeated in 2015.

### 4.9. Selection of Potential Defense-Related Genes

Samples of the two lines (“PI 201234” and “Qiemen”) were collected at three time points (6 h, 24 h, and 5 days) after inoculating with a zoospore suspension or sterile water. Total RNA was extracted and cDNAs were reverse transcribed from 2 ug of total RNA as described above. Next, qPCR was used to validate the expression of some DEGs identified by RNA-seq, with *actin* used as an internal control. All treatments were performed with three biological replicates, and each qRT-PCR experiment was repeated three times. The experiment was performed in 2014 and repeated in 2015.

## 5. Conclusions

In this study, transcriptome changes in the resistant line “PI 201234” were examined after *P. capsici* inoculation by RNA-seq. Among the 1220 DEGs detected, 480 were up-regulated, and 740 were down-regulated. The expression levels of 12 defense-related DEGs in resistant “PI 201234” and susceptible “Qiemen” were evaluated by qPCR. Furthermore, the expression patterns of these genes at different time points were significantly affected following *P. capsici* infection. Seven DEGs that are likely to be defense-related genes were also discussed and may play important roles in combating exogenous pathogens. In subsequent studies, the primary functions of the defense-related genes described in this paper will be examined. The RNA-seq data and analysis presented in this paper will be helpful for revealing the regulatory mechanisms of pepper resistance to *P. capsici.*

## Supplementary Materials

Supplementary materials can be found at http://www.mdpi.com/1422-0067/16/05/11417/s1.
